# Adapting Semi-Active Prostheses to Real-World Movements: Sensing and Controlling the Dynamic Mean Ankle Moment Arm with a Variable-Stiffness Foot on Ramps and Stairs

**DOI:** 10.3390/s21186009

**Published:** 2021-09-08

**Authors:** Jennifer K. Leestma, Katherine Heidi Fehr, Peter G. Adamczyk

**Affiliations:** 1 Department of Mechanical Engineering, University of Wisconsin-Madison, Madison, WI 53706, USA; kfehr@wisc.edu (K.H.F.); peter.adamczyk@wisc.edu (P.G.A.); 2 Department of Biomedical Engineering, University of Wisconsin-Madison, Madison, WI 53706, USA; 3 George W. Woodruff School of Mechanical Engineering, Georgia Institute of Technology, Atlanta, GA 30332, USA; 4 Institute for Robotics and Intelligent Machines, Georgia Institute of Technology, Atlanta, GA 30332, USA

**Keywords:** prosthesis, locomotion mode, wearables, gait biomechanics, semi-active device, prosthetic control

## Abstract

(1) Background: Semi-active prosthetic feet can provide adaptation in different circumstances, enabling greater function with less weight and complexity than fully powered prostheses. However, determining how to control semi-active devices is still a challenge. The dynamic mean ankle moment arm (DMAMA) provides a suitable biomechanical metric, as its simplicity matches that of a semi-active device. However, it is unknown how stiffness and locomotion modes affect DMAMA, which is necessary to create closed-loop controllers for semi-active devices. In this work, we develop a method to use only a prosthesis-embedded load sensor to measure DMAMA and classify locomotion modes, with the goal of achieving mode-dependent, closed-loop control of DMAMA using a variable-stiffness prosthesis. We study how stiffness and ground incline affect the DMAMA, and we establish the feasibility of classifying locomotion modes based exclusively on the load sensor. (2) Methods: Human subjects walked on level ground, ramps, and stairs while wearing a variable-stiffness prosthesis in low-, medium-, and high-stiffness settings. We computed DMAMA from sagittal load sensor data and prosthesis geometric measurements. We used linear mixed-effects models to determine subject-independent and subject-dependent sensitivity of DMAMA to incline and stiffness. We also used a machine learning model to classify locomotion modes using only the load sensor. (3) Results: We found a positive linear sensitivity of DMAMA to stiffness on ramps and level ground. Additionally, we found a positive linear sensitivity of DMAMA to ground slope in the low- and medium-stiffness conditions and a negative interaction effect between slope and stiffness. Considerable variability suggests that applications of DMAMA as a control input should look at the running average over several strides. To examine the efficacy of real-time DMAMA-based control systems, we used a machine learning model to classify locomotion modes using only the load sensor. The classifier achieved over 95% accuracy. (4) Conclusions: Based on these findings, DMAMA has potential for use as a closed-loop control input to adapt semi-active prostheses to different locomotion modes.

## 1. Introduction

Current passive prosthetic feet are offered in a variety of stiffness categories that are determined based on the user’s body weight. This prescription method results in prominent stiffness variability within single categories, both across manufacturers and even within single manufacturers [1,2,3,4]. Since these passive feet have only a single stiffness profile, the foot cannot change properties across walking speeds or locomotion modes, such as walking up or down ramps and stairs [5]. However, previous work has shown that healthy individuals actively regulate foot and ankle biomechanical properties across both speed [6,7,8] and locomotion mode [6,7,8,9,10,11,12,13]. Thus, current prostheses may limit a user’s locomotion due to their inability to reproduce this natural adaptation.

To optimize the walking performance of persons with amputation, prosthetic foot properties need to adapt. Active control through a powered prosthetic foot may provide this capability, but the added weight, height, power requirements, and cost of such devices present barriers to adoption [14]. An alternative is semi-active prostheses, which use minimal actuation to adjust device mechanical properties but do not power the user’s movement. Researchers have used this concept to create variable-stiffness prosthetic feet that can actively modulate their stiffness during the swing phase of walking [15,16,17,18,19]. These recent prosthetic developments have the potential to improve ambulation of persons with amputation by adapting stiffness to match locomotion speeds and modes, such as level ground, slopes, or stairs.

However, determining how to control stiffness adaptation is a challenge. Changes could be controlled using open-loop mapping—e.g., predetermined stiffness for every speed and locomotion mode—or using closed-loop adaptation to achieve some biomechanical outcome. For the closed-loop case, an appropriate target measure is needed that can be measured in real time using onboard sensors. Various biomechanical measures could fill this void, such as rollover shape [5,20], ankle impedance [21,22,23], or human foot quasi-stiffness [9,24]. However, these measures vary throughout the stance phase, making them more appropriate for fully powered prostheses; semi-active devices that can adjust only once per stride require a control target that summarizes ankle biomechanics across the whole stance phase in a single value.

The dynamic mean ankle moment arm (DMAMA) measure [8] was developed to be such a summary metric. DMAMA computes the ratio of the sagittal ankle moment impulse to the ground reaction force impulse across the whole stance phase. This measure has units of length, and its value represents the mean moment arm of the ground reaction impulse in front of the ankle. DMAMA quantifies the net dynamic effects of the ground reaction force on the ankle joint, resulting in a single measure that varies across behaviors such as walking vs. running and speed changes [8]. It is related biomechanically to the interaction of ankle angle and stiffness [8]. DMAMA is conceptually and computationally simple and is therefore well suited as a closed-loop target to control semi-active prosthesis stiffness for biomimetic adaptation to different behaviors. However, no methods have been established to enable the calculation of DMAMA using prosthesis-embedded sensors, which is necessary for closed-loop control.

Biomimetic closed-loop control of DMAMA using a semi-active prosthesis requires both the ability to vary DMAMA (e.g., through varying the prosthetic stiffness or ankle angle) across locomotion modes (level ground, ramps, and stairs) and the ability to estimate the locomotion mode in real time to permit such adaptation [8]. Previous work has established the validity of locomotion mode classification for transfemoral [25,26] and transtibial [27,28] prostheses. However, these applications frequently require several sensors, including load cell, inertial measurement unit, and/or electromyography data [25,29]. To better the probability of clinical adoption, ideal solutions would require limited sensors and use sensors that can be embedded in the prosthesis. Because the DMAMA calculation inherently requires a load cell sensor, an ideal locomotion mode classifier would be based only on load signals and would not require any additional sensors. However, it is unclear whether the locomotion mode of individuals with a transtibial amputation can be accurately detected using only a prosthesis-embedded load cell.

This study explores the potential for DMAMA to be used as a real-time control input for semi-active prosthetic devices. We aim to understand how DMAMA can be influenced by changes in prosthesis forefoot stiffness and by locomotion mode, specifically level ground, ramps, and stairs. We further investigate whether it is feasible to use a universal control law to exploit these relationships for control, or whether subject-dependent control laws are necessary, by evaluating subject-dependent and subject-independent trends across stiffness and locomotion modes. Additionally, we explore what it would take to employ such a measure in a real time, “outside of the lab” environment. We demonstrate a method to monitor both DMAMA and locomotion mode outside of the lab using only a prosthesis-embedded load cell sensor.

In this work, we first investigate whether stiffness or locomotion mode has a significant effect on DMAMA. We then evaluate how well overall trends can explain intra-subject DMAMA fluctuations. Our first hypothesis is that there will be a positive linear sensitivity of DMAMA to forefoot stiffness across all locomotion modes (i.e., a stiffer forefoot will cause DMAMA to be farther in front of the ankle). Our second hypothesis is that there will be a positive linear sensitivity of DMAMA to the ground incline across all stiffnesses (i.e., slope increasing from downhill to uphill will cause DMAMA to be farther in front of the ankle). Finally, we use linear discriminant analysis (LDA) to test the classification accuracy of locomotion modes using only prosthesis geometric measurements and the inputs from a six-axis pylon-embedded load cell. Throughout this work, we discuss how DMAMA is affected by changes in stiffness and locomotion mode and evaluate the potential of this measure to be used in real-time control of semi-active prosthetic devices.

## 2. Materials and Methods

### 2.1. Participants

Four adult participants with transtibial amputation (a subset of a larger study) participated in this focused test of the prosthesis-embedded technology. These participants (4 males; weight 90 ± 15 kg; height 1.815 ± 0.15 m) wore a novel prosthetic foot and a suite of prosthesis-embedded and wearable sensors that recorded the signals necessary for field-based analysis of prosthetic limb mechanics. Each participant used a prosthesis for daily ambulation and walked without the help of an ambulatory aid. Prior to beginning the study, each participant signed a written consent form approved by the University of Wisconsin–Madison Health Sciences institutional review board.

### 2.2. Experimental Protocol

For this study, prosthetic forefoot stiffness variation was supplied by the variable-stiffness foot (VSF), a custom-built prosthesis previously published by Glanzer and Adamczyk [16]. The participants did not wear a foot shell or shoe on the prosthetic foot and wore their chosen athletic shoe on their unaffected foot. The height of the VSF was adjusted by a prosthetist to ensure proper bilateral alignment in the absence of a prosthetic-side shoe. Use of the VSF ensured that prosthesis alignment was consistent across trials and that stiffness profiles were consistent across participants. This prosthetic foot uses a small motor to adjust the free length of a cantilever forefoot spring, thereby altering forefoot stiffness. The study evaluated three different prosthetic forefoot stiffnesses that were scaled to the participants’ body weight and comfort levels. We set the low stiffness value to the minimum stiffness permitted by each participant’s body mass, ensuring that the prosthetic keel deflection did not reach its physical limit. We set the high stiffness value to the VSF’s maximum-possible stiffness of 32 N/mm, determined through mechanical testing (Model 120-P-1000; TestResources, Shakopee, MN, USA). The medium stiffness value was set at the mean of the low and high stiffnesses.

**Figure 1:**
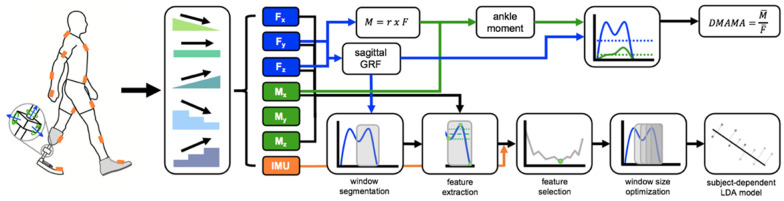
Collection and calculation using wearable sensors. Participants wore an IMU suit (orange) and a 6-axis load cell, which read forces (blue) and moments (green). Participants were then asked to walk across level ground, ramps, and stairs. The IMU suit and force data collected were used both to calculate DMAMA and to act as inputs to the machine learning model.

We instructed participants to walk through a circuit in a campus building that required five locomotion modes: level ground, ramp ascent (+5° incline) and descent (−5° incline), and stair ascent and descent (178 mm step height, 311 mm anteroposterior tread depth, 40 mm step overhang, two sequential flights of 12 and 13 stairs, up and then down). All participants walked this circuit once using each of three stiffness conditions. The participants walked the course at their preferred walking speed, as the goal of the study was to see how participants would ambulate with various prosthesis stiffnesses in a real-world environment. The circuit resulted in 50 ± 14 level ground, 11 ± 3 up ramp, 11 ± 3 down ramp, 11 ± 1 up stairs, and 11 ± 1 down stairs strides per trial that were used for analysis. Transition steps between locomotion modes were excluded from this analysis. This experimental setup can be viewed in Figure 1.

### 2.3. Portable Data Collection Methods

The calculation of DMAMA requires the sagittal ground reaction force (GRF) and ankle moment. In a gait laboratory, these measures are typically found from force plate and motion capture data [8]. However, this equipment is not portable, inhibiting applications in environments outside of a lab. Instead, we calculated DMAMA on the side of amputation using a six-axis load cell (iPecs; RTC Electronics, Dexter, MI, USA) embedded in the prosthetic pylon immediately below the participant’s daily-use prosthetic socket. The load cell collected at a frequency of 149.9 ± 38.5 Hz (mean ± SD); this variability was accounted for by using measured sample time in all calculations. The validity of the load cell for lower-limb prosthetics research has been previously established [30,31]. The VSF prosthesis [16] was installed beneath the load cell. We then had a certified prosthetist perform a standard prosthetic alignment with the VSF in a medium-stiffness condition.

We fit the participants with a full-body set of inertial measurement units (IMUs) (MVN Awinda; Xsens B.V., Enschede, The Netherlands) that was used to discern changes in locomotion mode during the trials. We calibrated a kinematic skeleton model reconstructed from IMU data (XSens MVN Analyze software) using each participant’s anthropometric measurements along with standing and walking calibration trials.

We used the load cell measurements as an approximation for the ground reaction force and moments in the sagittal plane. Prior to beginning the study, we measured the orientation and location of the load cell relative to the user’s prosthetic pylon and body segments using motion capture (12-camera OptiTrack Prime13 system; NaturalPoint Inc., Corvallis, OR, USA) in the laboratory. Functional movement calibration trials were used to calibrate the model in Visual3D, allowing the determination of the biological joint centers. We determined functional joint centers for the knee and hip from the motion capture trials using the Gillette algorithm (Visual3D software; C-Motion Inc., Germantown, MD, USA). The ankle joint center for the prosthetic side was chosen as a fixed physical location on the prosthesis comparable to the biological limb, as there is no fixed prosthetic ankle rotational axis. It should be noted that this method of defining the prosthetic ankle joint axis reduces across subject variability but may result in an offset error relative to a biological DMAMA measure. The joint centers’ positions relative to the center of the load cell were determined using the average across the function movement trial. This step was taken to orient the load cell forces in the pylon/shank reference frame prior to all calculations. We recorded the locations of the knee and ankle joints relative to the load cell for use in joint moment calculations.

### 2.4. Calculating DMAMA from Prosthesis-Embedded Load Cell Data

We measured the force and moment about the load cell origin directly from the load cell embedded in the prosthetic pylon. To correct the load cell’s orientation and placement relative to the participant’s shank, we found the rotation matrix (${\bar{R}}_{LC}^{Shank}$) that transforms the load cell’s (LC) coordinate system into a shank-based coordinate system using motion capture data. We determined the orientation and position of the load cell based on the 3D coordinates of the three markers placed on it. We found the final rotation matrix for each participant by calculating the load-cell-to-shank-frame rotation matrices for 300 samples. The rotation matrices were converted to rotation vectors using Rodrigues’ rotation formula. The 300 vectors were averaged element-wise and converted into a single definitive rotation matrix. Prior to DMAMA and moment calculations, we multiplied the force and moment data reported by the load cell by the calculated rotation matrix (${\bar{R}}_{LC}^{Shank}$). We also determined the vectors from the knee and VSF ankle joint to the load cell center in a similar manner, calculating them for 300 samples and finding the average.

We determined joint moments about the ankle and knee, ${\vec{M}}_{ankle}$ and ${\vec{M}}_{knee}$, using quasi-static vector mechanics according to

(1) $$\vec{M}={\vec{r\;}}_{LC}^{}\times {\vec{F}}_{sagittal}+{\vec{M}}_{sagittal}$$

where ${\vec{r}}_{LC}^{}$ is the vector from the joint center to the center of the load cell, ${\vec{F}}_{sagittal}$ is the load cell force vector in the sagittal plane, and ${\vec{M}}_{sagittal}$ is the load cell moment in the sagittal plane. This equation neglects inertial terms due to low stance-phase segment accelerations. This equation was used to calculate both the ankle and knee joint sagittal plane moments using the appropriate vector ${\vec{r}}_{LC}^{}$ from each joint to the load cell. We then calculated the DMAMA value as the ratio of the sagittal ankle moment impulse (extensor positive) to the magnitude of the sagittal ground reaction force impulse for each individual stance phase, according to

(2) $$DMAMA=\frac{J}{I}=\frac{\int_{HS}^{TO}{\vec{M}}_{ankle}\;dt}{\left||\int_{HS}^{TO}{\vec{F}}_{sagittal}\;dt|\right|}=\frac{{\bar{M}}_{ankle}}{{\bar{F}}_{sagittal}}$$

where J is the magnitude of ankle moment impulse, I is the magnitude of the GRF impulse, ${\vec{M}}_{ankle}$ is the ankle moment in the sagittal plane, ${\vec{F}}_{sagittal}$ is the load cell force vector in the sagittal plane, ${\bar{M}}_{ankle}$ is the mean ankle moment in the sagittal plane, and ${\bar{F}}_{sagittal}$ is the magnitude of the mean load cell force vector in the sagittal plane. We normalized DMAMA to a percentage of the VSF foot length. We performed these calculations with a custom MATLAB script (MATLAB 2019a; Mathworks, Inc.; Natick, MA, USA). This processing pipeline can be viewed in Figure 1.

### 2.5. Machine Learning Model: Determining the Locomotion Mode from Load Cell Data

We investigated the use of a machine learning model to determine how well data from the pylon load cell alone could discriminate among different locomotion modes, with the goal of using the locomotion mode in an eventual control algorithm. For the model, we used nine sensor inputs (${\vec{F}}_{x}$, ${\vec{F}}_{y}$, ${\vec{F}}_{z}$, ${\vec{M}}_{x}$, ${\vec{M}}_{y}$, ${\vec{M}}_{z}$, ${\vec{M}}_{ankle}$,$\;{\vec{M}}_{knee}$,$\;{\vec{F}}_{sagittal}$). Prior to building the model, all inputs were resampled to 100 Hz to compensate for the non-uniform data collection frequency of the load cell. We performed feature extraction once per gait cycle from a data window preceding toe-off, with toe-off detected by the sagittal force falling below 8% body weight. We ended the window at toe-off because it is easily detectable using a prosthesis load cell and includes information from the stance phase prior to making a decision on how to control stiffness in the swing phase. For each sensor input, we extracted a feature set consisting of the mean, standard deviation, minimum value, maximum value, starting value, and ending value within the data window [25,32,33]. We separated feature sets by stiffness settings of the VSF (low, medium, high), as stiffness would be known in the case of real-time control. We used linear discriminant analysis (LDA) to create subject- and stiffness-dependent models. We used 10-fold validation to accommodate the small data set. For the model, we performed forward feature selection using a 30 ms window of data prior to toe-off. The chosen feature set included the features that resulted in the minimum classification error during the forward feature selection process. Next, we optimized the window size of the reduced-feature model, testing 100–500 ms windows in 33 ms increments. The model was then finalized using the feature set determined from forward feature selection and window size optimization processes. This process can be viewed in Figure 1.

### 2.6. Statistics

Subject-independent data provide an understanding of how DMAMA is influenced by stiffness and locomotion mode across all subjects. To further evaluate the efficacy of DMAMA as a control strategy, we analyzed how well subject-independent trends explain intra-subject variations in DMAMA. A strong correlation between subject-independent trends and intra-subject data would suggest that a subject-independent control strategy may be feasible; however, a poor correlation may suggest that a subject-dependent controller is the needed approach.

We evaluated the effects of both stiffness and ground incline on DMAMA. We first evaluated subject-independent relationships using a linear mixed-effects model where either the stiffness or the ground incline was the fixed effect and the different participants were random effects. We separated the data by locomotion mode and ran a linear regression on the mean DMAMA values across participants and stiffness settings, allowing subject-dependent intercepts to account for the random effect. We determined the subject-independent sensitivity of DMAMA to each variable (slope of the best-fit line), the statistical significance of sensitivity (*p*-value being different from zero), and the amount of variance explained by the model (R^2^).

Next, we evaluated subject-dependent relationships between stiffness and DMAMA and between ground incline and DMAMA. We ran a linear regression on each individual participant’s data and determined the subject-dependent sensitivity of DMAMA to each variable (slope of the best-fit line), the statistical significance of sensitivity (*p*-value being different from zero), and the amount of variance explained by the model (R^2^). We also evaluated the correlation strength of the subject-independent best-fit line on each participant’s stride-by-stride data to determine how well the overall (subject-independent) best-fit line explained the variance in individual participants’ data. To represent this, we calculated the R^2^ value using

(3) $$R^{2}=1-\frac{RSS}{TSS}=1-\frac{\sum {\left(DMAMA_{adjusted}-DMAMA_{predicted}\right)}^{2}}{\sum {\left(DMAMA_{adjusted}-DMAMA_{average}\right)}^{2}}$$

where RSS is the residual sum of squares, TSS is the total sum of squares, $DMAMA_{adjusted}$ is the DMAMA value calculated for each step and adjusted for the random effect, $DMAMA_{predicted}$ is the DMAMA value predicted by the linear mixed-effects model, and $DMAMA_{average}$ is the average adjusted DMAMA value for the individual participant.

Across all analyses, we set α = 0.05 to determine statistical significance. Statistical analyses were performed using Origin 2020 (OriginLab Corporation, Northampton, MA, USA) and MATLAB 2019a (Mathworks, Inc.; Natick, MA, USA).

## 3. Results

### 3.1. Sensitivity of DMAMA to Stiffness across Ground Incline and Stairs

**Figure 2:**
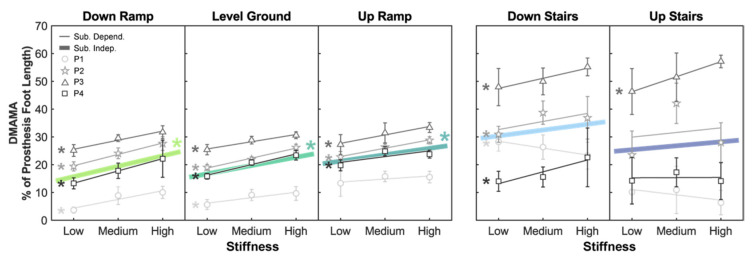
Linear trends showing the effect of prosthesis forefoot stiffness on DMAMA. Colored lines show the subject-independent data fit as a result of the linear mixed-effects model. Gray markers and vertical bars show each participant’s average and standard deviation values, respectively. Gray lines show subject-dependent linear fits. The asterisk (*) indicates a linear trend with a significant *p*-value (*p* < 0.05).

We first evaluated the subject-independent sensitivity of DMAMA to stiffness in each of the locomotion modes: down ramp (DR), level ground (LG), up ramp (UR), down stairs (DS), and up stairs (US) (Figure 2). There was a significant positive sensitivity of DMAMA to stiffness (*p* < 0.05) in the DR, LG, and UR conditions (sensitivity range: 2.299–3.749 percent foot length per stiffness increment) but not in the DS (*p* = 0.124) or US (*p* = 0.522) condition (Table 1 and Table 2).

We then evaluated the subject-dependent sensitivity of DMAMA to stiffness in each of the locomotion modes (Figure 2). There was a positive sensitivity (*p* < 0.05) in all four participants in the DR condition (sensitivity range: 3.132–4.422 percent foot length per stiffness increment), all four participants in the LG condition (1.963–3.824 percent foot length per stiffness increment), three of four participants in the UR condition (2.070–3.032 percent foot length per stiffness increment), three of four participants in the DS condition (2.906–4.263 percent foot length per stiffness increment), and one of four participants in the US condition (sensitivity: 5.405 percent foot length per stiffness increment) (Table 3). There was a negative sensitivity (*p* < 0.05) of DMAMA to stiffness for one participant in the DS condition (−2.479 percent foot length per stiffness increment).

### 3.2. Sensitivity of DMAMA to Ground Incline across Stiffnesses

**Figure 3:**
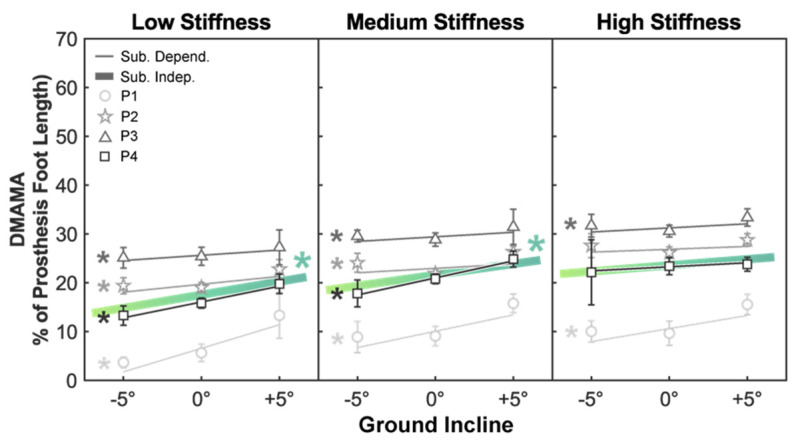
Linear trends showing the effect of the ground incline on DMAMA. Colored lines show the subject-independent data fit as a result of the linear mixed-effects model. Gray markers and vertical bars show each participant’s average and standard deviation values, respectively. Gray lines show subject-dependent linear fits. The asterisk (*) indicates a linear trend with a significant *p*-value (*p* < 0.05).

Next, we evaluated the subject-independent sensitivity of DMAMA to the ground incline across forefoot stiffness (Figure 3). Across all participants, there was a positive sensitivity (*p* < 0.05) of DMAMA to the ground incline in the low- (sensitivity: 0.540 percent foot length per degree incline) and medium- (0.450 percent foot length per degree incline) stiffness conditions but not in the high-stiffness condition (Table 4 and Table 5).

We then evaluated the subject-dependent sensitivity of DMAMA to the ground incline across forefoot stiffness (Figure 3). There was a positive sensitivity (*p* < 0.05) in all four participants in the low-stiffness condition (sensitivity range: 0.214–0.965 percent foot length per degree incline), all four participants in the medium-stiffness condition (0.181–0.699 percent foot length per degree incline), and two of four participants in the high-stiffness condition (0.169–0.535 percent foot length per degree incline) (Table 6).

### 3.3. Multivariate Regression of DMAMA to Stiffness and Incline

**Figure 4:**
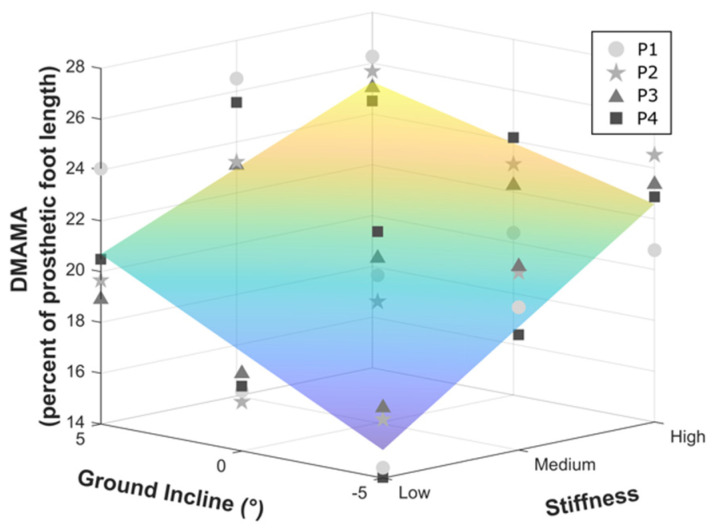
Multivariate regression on the combined effects of stiffness and ground incline on DMAMA.

Considering only the ground incline conditions, the sensitivity of DMAMA to stiffness was the greatest in the DR condition (sensitivity: 3.749 percent foot length per stiffness increment), moderate in the LG condition (2.993 percent foot length per stiffness increment), and the least in the UR condition (2.299 percent foot length per stiffness increment). Similarly, the sensitivity of DMAMA to the ground incline was the greatest in the low-stiffness condition (0.540 percent foot length per degree incline), moderate in the medium-stiffness condition (0.450 percent foot length per degree incline), and the least in the high-stiffness condition (0.250 percent foot length per degree incline). We formalized this interaction by fitting a multiple linear regression with incline, stiffness, and incline-times-stiffness interaction terms to the DMAMA data for the three ground incline conditions and three stiffnesses (Figure 4). This multivariate regression revealed positive coefficients for sensitivity to stiffness (coefficient: 3.01, CI: ±0.744 percent foot length per unit stiffness) and incline (coefficient: 0.413, CI: ±0.149 percent foot length per degree incline) and a negative sensitivity to their interaction (coefficient: −0.145, CI: ±0.182).

### 3.4. Accuracy of Gait Mode Classification from a Prosthesis-Embedded Load Cell

**Figure 5:**
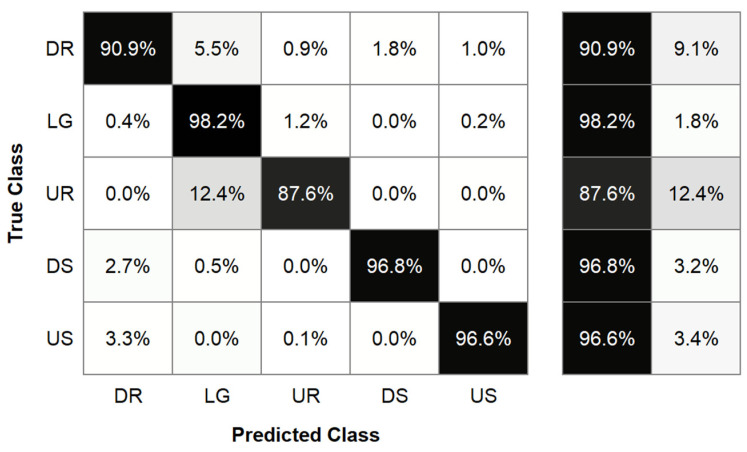
Confusion matrix results from a linear discriminant analysis locomotion mode classifier. The true class is shown on the left axis, indicating the correct locomotion mode. The predicted class is shown on the bottom axis, indicating the locomotion mode predicted by our classifier. The table to the right of the confusion matrix shows the percentages of strides that were correctly (left) and incorrectly (right) classified by our model.

We then evaluated the accuracy of the subject-dependent LDA classifier. After the initial input of 54 features extracted from a 300 ms window, forward feature selection led to the elimination of 32 features, leading to a model that used 22 features. We then optimized the window size, leading to the selection of a 300 ms window. This final subject- and stiffness-dependent LDA model accurately classified 90.9% of down ramp steps, 98.2% of level ground steps, 87.6% of up ramp steps, 96.8% of down stairs steps, and 96.6% of up stairs steps (Figure 5). This led to an overall model accuracy of 95.72%. It should be noted that this model used imbalanced classes for training and testing, with the majority class being level ground.

### 3.5. Calculation of Joint Moments Using a Prosthesis-Embedded Load Cell

**Figure A1:**
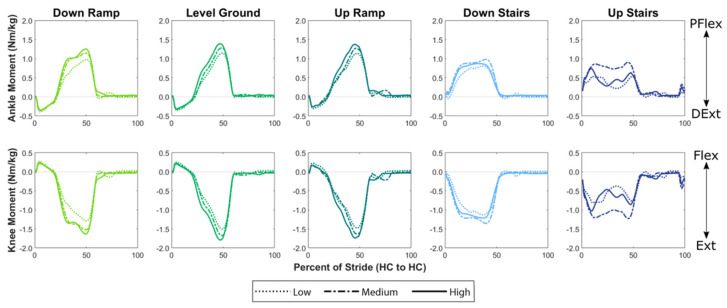
Ankle and knee moment throughout the gait cycle across stiffness for each locomotion mode. These graphs show the average moment profiles across all trials for a representative subject.

**Figure A2:**
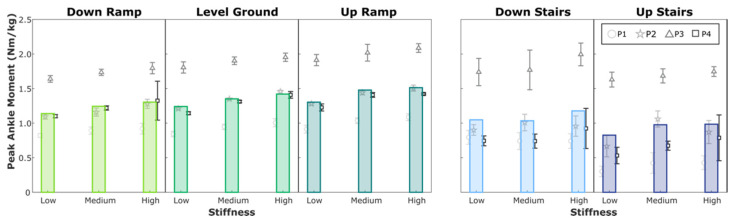
Peak ankle plantarflexion moment across stiffness for each locomotion mode. Colored bars show the subject-independent average. Gray markers and vertical bars show each participant’s average and standard deviation values, respectively.

**Figure A3:**
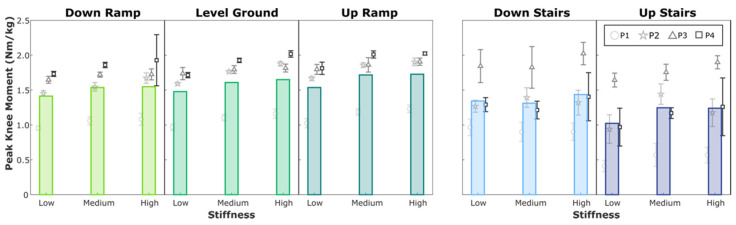
Peak knee extension moment across stiffness for each locomotion mode. Colored bars show the subject-independent average. Gray markers and vertical bars show each participant’s average and standard deviation values, respectively.

**Figure A4:**
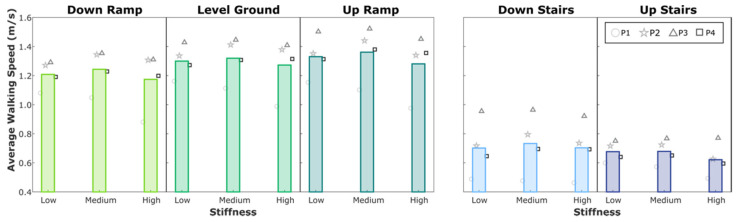
Average walking speed across stiffness for each locomotion mode. Walking speed was not controlled for in this study. Colored bars show the subject-independent average walking speed for each condition. Gray markers show each participant’s average walking speed.

Because DMAMA is a summary measure computed from traditional joint mechanics data, these joint mechanics are also available within the analysis. We provide these data as an illustrative supplement in Appendix A, including graphs of both knee and ankle moments for a representative participant as time series plots (Figure A1), peak ankle moment (Figure A2), peak knee moment (Figure A3), and walking speed (Figure A4).

## 4. Discussion

### 4.1. Methods for “Outside of the Lab” DMAMA Calculation from a Load Cell Sensor

Biomimetic closed-loop control of semi-active prostheses demands a biomechanical control target that can be measured and manipulated in environments “outside of the lab.” DMAMA is a suitable metric to drive semi-active control due to simplicity and can be measured in real-world scenarios. In this work, we established a method to calculate DMAMA from only a pylon-embedded load cell sensor and prosthesis geometric measurements, eliminating the need for force plates and motion capture systems. Prior work has established that wearable technology (specifically, pressure-measuring insoles measuring intact limbs) could evaluate trends in DMAMA with similar sensitivity to laboratory-based measurements. The present work extends this to approximate the ideal case of a direct force and moment measurement fully embedded in the device that uses the data. Both these cases relied on approximations of the location of the ankle joint relative to the sensor, introducing potential offset errors in DMAMA. In contrast, future implementation of a load cell directly embedded in an ankle-foot prosthesis would result in specified, rather than measured, geometric relationships between the load cell and the ankle and would therefore eliminate the approximation steps required in this work and reduce variability and uncertainty in the measurements.

### 4.2. Relationship between DMAMA and Stiffness

The results suggest a significant positive sensitivity of DMAMA to forefoot stiffness in down ramp, level ground, and up ramp conditions but no clear effect on stairs due to high variability. Additionally, the linear fit showed a strong correlation between stiffness and DMAMA in the down ramp, level ground, and up ramp conditions. Within the ramp conditions, the significant sensitivity of DMAMA to stiffness varied across the ground incline, with DMAMA being less sensitive to stiffness as the incline increased. This leads us to partially accept our first hypothesis (increase in DMAMA with stiffness): DMAMA does increase with forefoot stiffness in walking-like conditions but perhaps not on stairs. The high variability of DMAMA on stairs may reflect qualitatively different stair ambulation strategies across individuals, which have made it difficult to study this behavior in other real-world studies as well [13].

We next looked to understand how well subject-independent sensitivity trends could explain individual participants’ DMAMA values. In down ramp, level ground, and up ramp conditions, the subject-independent sensitivity provided a moderate-to-strong fit for individual participant data, while it provided a weak fit in the down stairs and up stairs conditions. No locomotion mode showed that a generalized sensitivity provided a strong fit for all participants. Even in the ramp and level ground conditions, where all individual participants had a significant positive sensitivity, participants had differing levels of sensitivity. Thus, we conclude that increasing forefoot stiffness can increase DMAMA in all participants, but the quantitative sensitivity is subject dependent.

### 4.3. Relationship between DMAMA and Ground Incline

The results also suggest a significant positive sensitivity of DMAMA to the ground incline in the low-and medium-stiffness settings but not in the high-stiffness setting. Additionally, the linear fit showed a strong correlation between ground incline and DMAMA in the low-stiffness condition and moderate correlations in the medium- and high-stiffness conditions. As expected, because of similar interaction trends seen in the stiffness analysis, the sensitivity to the incline decreases as stiffness increases. This leads us to partially accept our second hypothesis: DMAMA does increase with the ground incline but with reducing sensitivity at higher stiffness settings.

We again looked to understand how well subject-independent sensitivity of DMAMA to the ground incline could explain individual participants’ DMAMA values. All participants exhibited a positive sensitivity of DMAMA to the ground incline. The goodness of fit of the subject-independent sensitivity to individual participants was highly variable across participants and ground inclines. Across all stiffness conditions, the subject-independent sensitivity provided some explanations of trends seen in participants 1 and 4, though the goodness of fit was highly variable. However, the subject-independent sensitivity provided a poor explanation of trends seen in participants 2 and 3. Therefore, as above we conclude that increasing the ground incline can increase DMAMA in all participants, but the quantitative sensitivity is subject dependent.

### 4.4. Interaction between Stiffness and Ground Incline

Some of the effects above could be explained by the interaction of our two independent variables, stiffness and ground incline. Our multiple regression revealed a negative best-fit coefficient for the interaction between stiffness and ground incline, though the coefficient did not reach the threshold for statistical significance. Nevertheless, an interaction with a negative sign could explain the trends observed above: that sensitivity of DMAMA to either independent variable is reduced at higher values of the other. This interaction can be thought of as a kind of saturation, as opposed to compounding (interaction with a positive sign). This effect makes sense mechanistically: both a stiffer forefoot and an uphill slope move the center of pressure toward the toe and lead to earlier heel lift-off and toe-only support, such that a high value of either leaves little room for additional forward movement due to the other. It is possible that avoiding such situations is itself a valuable goal in prosthetic ankle control; if so, a controller that reduces stiffness on uphill slopes and increases stiffness on downhill slopes would serve to keep DMAMA lower in support of this goal.

### 4.5. Interpretation of DMAMA Changes with Stiffness and Ground Incline

The positive sensitivity of DMAMA to stiffness could be explained if participants maintain similar leg kinematics regardless of forefoot stiffness. If leg kinematics remain constant, stiffening of the forefoot will cause a more rapid forward shift of the COP during foot roll-over, therefore increasing the DMAMA value. The observed variation in DMAMA with stiffness suggests that persons with amputation may not change their kinematics to maintain the same DMAMA value when stiffness changes. This demonstrates that DMAMA can be varied in persons with amputation by manipulating stiffness. Direct evaluation of how well kinematics are retained is a component of the broader study from which these pilot data were collected and will be explored in future work.

### 4.6. Feasibility of DMAMA to Drive a Closed-Loop Biomimetic Controller

The moderate stride-by-stride fluctuations in DMAMA (standard deviation bars in Figure 2 and Figure 3) imply that a controller should not adjust the prosthesis stiffness in response to rapid changes in DMAMA measurements, as both the DMAMA value and the predicted effect of a stiffness change have too much residual variability. However, the generalized sensitivity to stiffness and incline was effective in explaining trends in participants’ mean DMAMA values, meaning that participants’ average behavior was well-characterized by the sensitivity. Thus, a control system could make meaningful adjustments by evaluating a moving average DMAMA value over several steps, rather than by just evaluating the previous step, when making a stiffness change. This slower-adapting control strategy would drive the controller with a data point more representative of steady-state behavior rather than individual step behavior. It would also be more stable and robust in the face of isolated fluctuations and therefore would likely be preferable to subjects who demand predictability from their prostheses. It could be overridden or reset in cases where a qualitative behavioral change would benefit from a rapid open-loop adjustment, such as switching to stairs, turning around on a slope, or running.

### 4.7. Subject-Independent vs. Subject-Dependent DMAMA Targets

The limited ability of subject-independent trends to fit individual participants’ data inhibits but does not necessarily preclude the ability to use a subject-independent control architecture using DMAMA. Subject-independent trends with stiffness and incline were correct in sign for all four participants, suggesting that a generalized controller would satisfy them all qualitatively, even if slightly mismatched from their preferred changes. However, both the offset in DMAMA (intercept of the linear fit, or the subject’s mean value) and the behavior in up stairs vs. down stairs conditions were highly subject dependent. Thus, some level of subject-dependent tunability in a DMAMA-based controller would likely be beneficial—for example, adjusting the range and sensitivity of the target DMAMA setting to variations in slope.

### 4.8. Locomotion Mode Classification

To determine whether real-time detection of the locomotion mode was possible with limited instrumentation, we evaluated the ability of an LDA classification algorithm to detect the locomotion mode based only on measures from a pylon-embedded load cell and shank segment geometric measurements. Our results showed that the LDA model was able to accurately classify locomotion mode for 95.72% of steps. The LDA model feature selection process revealed which sensors and signal features were the most useful in determining the locomotion mode. This selection process eliminated all features that were derived from the sagittal force, but kept features derived from the remaining eight sensor inputs. The selection process kept all feature types (such as mean or starting value), indicating that all feature types provided some value in determining the locomotion mode. The model’s selection of two features that were derived from sensor input that required some calculation before passing into the model (${\vec{M}}_{ankle}$,$\;{\vec{M}}_{knee}$) revealed that subject-dependent prosthesis geometric measurements and biomechanically meaningful preprocessing contribute to accurate locomotion classification.

The LDA model disproportionately misclassified up ramp (UR) trials in comparison to other classes, where up ramp misclassifications were primarily classified as level ground (LG) steps. We expect that this error is primarily attributed to the class imbalance in the data set, where we collected 50 ± 14 level ground and 11 ± 3 up ramp steps per participant. This class imbalance also explains why a comparable percentage of level ground steps are not misclassified as up ramp steps. Previous work has shown that the feature space between level ground and up ramp steps is similar, which may provide an explanation for the increased difficulty discerning these steps using our model [26,34]. Other studies of lower-limb prostheses have demonstrated the feasibility of combining level ground and up ramp classes, both for classifying locomotion modes and defining impedance parameters for a prosthesis [35,36]. Our finding of reduced sensitivity of DMAMA to stiffness agrees with this idea, as misclassifying up ramp steps as level ground steps would cause relatively little error in adjusting the prosthesis stiffness. This suggests that future applications of this model could combine level ground and up ramp classes, eliminating the concern for this misclassification.

This analysis was intended to investigate the feasibility of locomotion mode classification from a single sensor (i.e., pylon-embedded load cell) using a simple machine learning model. However, we believe that more advanced machine learning algorithms may be able to improve subject-dependent model accuracy or provide subject-independent classifiers [32]. Our results support the feasibility of using only a load cell sensor to classify locomotion modes in individuals with transtibial amputation, permitting the use of such a classifier in a closed-loop device controller that adjusts based on mode.

### 4.9. Addressing Hypotheses

The results of this study support both key hypotheses: that there would be (1) positive linear sensitivity of DMAMA to forefoot stiffness across all locomotion modes and (2) positive linear sensitivity of DMAMA to the ground incline across all stiffnesses. These results suggest that both stiffness and locomotion mode influence DMAMA, and therefore both variables need to be considered when targeting DMAMA changes. Stiffness can be considered in real time using the known stiffness value from the foot’s existing control system. Additionally, the locomotion mode can be determined using only a pylon-embedded load cell and prosthesis geometric measurements as inputs into a machine learning model. These results suggest that DMAMA could feasibly be used as a control parameter for a variable-stiffness foot and potentially for other semi-active prosthetic devices.

### 4.10. Future Directions

The core practical application of these findings is to use variations in stiffness to enable some level of adjustment of a prosthesis to the user’s behavior and the terrain. The sensitivity of DMAMA to stiffness was modest in this experiment, suggesting that this effect might be limited in its application. However, current prostheses allow no adaptability at all; if emerging technology can offer any benefit, it will be an improvement on the current experience of prosthesis users. Furthermore, the VSF was originally conceived to also adjust parameters such as energy storage and return from the prosthesis and stability in qualitatively different behaviors like standing vs. walking [16]; if continuous adjustments across locomotion modes or inclines is another addition on top of these substantial benefits, it will be worth including in the device’s final controller.

One additional use case for a controlled VSF could be a DMAMA-matching controller that seeks to match this metric on the prosthetic side to values measured in real time from the intact limb. Such measurements could be made using force-sensitive insoles [8] or even body-mounted wearables such as the recently developed technology of tendon tensiometry [13]. Alternatively, pre-mapped rules to determine DMAMA targets on different terrains could be used. The same control targets could also be used for powered prostheses, and in that case could rely on sensors internal to the prosthesis to determine the DMAMA estimate and directly control the ankle moment. Furthermore, the ability to estimate DMAMA from other sensors (not just pylon-embedded load cells) could enable extensions of DMAMA-based control to exoskeleton applications to improve compatibility of these systems with normal gait on different terrains.

### 4.11. Limitations

This study used a subset of data from a larger study to explore this concept of DMAMA as a possible real-time control variable. The study was limited by the small number of participants who used all the sensors required to compute DMAMA and track and label the different terrains. The small sample limited our ability to make conclusive claims about the biomechanical results of DMAMA. A study with a larger sample, perhaps also including tests that implement the controllers suggested by this analysis, could further explore subject-independent vs. subject-dependent control laws and classifiers and could establish an “existence proof” of the closed-loop control motivated through this study.

Each participant wore the same VSF for this study, preventing our ability to match the length of the VSF prosthesis to their prescribed prosthesis or intact foot. A practical implementation of a VSF in clinical use would need to accommodate multiple sizes of prostheses for users of different heights, but this was not possible with only one prototype.

Another potential limitation was the treatment of footwear in this study; participants wore their chosen athletic shoe on their biological foot and no shoe at all on the prosthesis. The use of preferred (rather than standardized) shoes could have affected the gait biomechanics across individuals in this study. Using a prosthesis with no shoe is uncommon in daily use but is common with certain high-performance prostheses (Versa Foot2, Biodapt, Saint Cloud, MN, USA) and running prostheses (Cheetah Xtend, Össur, Reykjavík, Iceland; Catapult Running Foot, Freedom Innovations, Irvine, CA, USA; Flex-Run, Össur, Reykjavík, Iceland). In future uses of advanced prostheses such as the VSF, it could become more common as users seek to maximize the function of their prostheses without letting a shoe affect its properties [37].

Lastly, this study allowed participants to walk at their preferred speeds across all conditions so that the tests would be ecologically valid relative to the eventual use of these results in uncontrolled real-world scenarios. Testing in these conditions provided us with information about how stiffness and ground incline changes would alter DMAMA in unconstrained environments; however, these results may be limited when evaluating how DMAMA is affected in speed-constrained studies or in conditions of varying speed. Additional studies of natural gait across locomotion modes or with combinations of speed and mode could build on prior findings of how DMAMA varies with speed on level ground [8].

## 5. Conclusions

In conclusion, we found that DMAMA has significant positive sensitivity to both stiffness and ground incline and negative sensitivity to their interaction. Sensor data from a pylon-embedded load cell and limited subject-dependent prosthesis geometric measurements provided sufficient information to compute DMAMA without external instrumentation and also to classify locomotion modes through a machine learning model. DMAMA may be a suitable practical control parameter for a semi-active variable-stiffness foot, enabling real-time alteration of prosthetic forefoot properties for improved biomechanics in comparison to passive prostheses.

## Figures and Tables

**Table 1:** Subject-independent regression slopes (versus stiffness) and descriptive statistics for DMAMA by locomotion mode. Linear mixed-effects model to explain variations in DMAMA with stiffness as the fixed effect and participants as the random effect. The linear fit for the effect of stiffness on DMAMA was generated for each locomotion mode separately. The sensitivity (percent foot length per stiffness increment), *p*-value, and R^2^ value are shown for each linear regression.

DOWN RAMP			LEVEL GROUND			UP RAMP			DOWN STAIRS			UP STAIRS		
Sensitivity to Stiffness	*p*-Value	R^2^	Sensitivity to Stiffness	*p*-Value	R^2^	Sensitivity to Stiffness	*p*-Value	R^2^	Sensitivity to Stiffness	*p*-Value	R^2^	Sensitivity to Stiffness	*p*-Value	R^2^
3.749	<0.001	0.993	2.993	<0.001	0.990	2.299	0.002	0.970	2.102	0.124	0.959	1.423	0.522	0.929

**Table 2:** Correlation strength of subject-independent model to subject-dependent data. Goodness of fit when using the subject-independent sensitivity of DMAMA to stiffness to explain individuals’ data; R^2^ values indicate how well subject-independent trends explained intra-subject variability.

		DOWN RAMP	LEVEL GROUND	UP RAMP	DOWN STAIRS	UP STAIRS
		R^2^				
Participant	1	0.512	0.248	–0.018	–0.479	–0.136
	2	0.734	0.849	0.705	0.138	0.014
	3	0.635	0.644	0.377	0.226	0.137
	4	0.428	0.790	0.355	0.166	–0.027

**Table 3:** Subject-dependent regression slopes (versus stiffness) and descriptive statistics for DMAMA by locomotion mode. Subject-dependent linear regression coefficients for the effect of forefoot stiffness on DMAMA in each locomotion mode, using individual participant data. The linear fit for the effect of stiffness on DMAMA is shown for each participant. The sensitivity (percent foot length per stiffness increment), *p*-value, and R^2^ value are shown for each linear regression.

		DOWN RAMP			LEVEL GROUND			UP RAMP			DOWN STAIRS			UP STAIRS		
		Sensitivity to Stiffness	*p*-Value	R^2^	Sensitivity to Stiffness	*p*-Value	R^2^	Sensitivity to Stiffness	*p*-Value	R^2^	Sensitivity to Stiffness	*p*-Value	R^2^	Sensitivity to Stiffness	*p*-Value	R^2^
Participant	1	3.132	<0.001	0.533	1.963	<0.001	0.344	1.092	0.085	0.078	–2.479	0.009	0.197	–1.915	0.147	0.067
	2	4.093	<0.001	0.740	3.587	<0.001	0.873	2.994	<0.001	0.745	2.906	0.022	0.149	1.668	0.504	0.016
	3	3.258	<0.001	0.650	2.596	<0.001	0.659	3.032	<0.001	0.400	3.627	0.001	0.274	5.405	<0.001	0.299
	4	4.422	<0.001	0.438	3.824	<0.001	0.829	2.070	<0.001	0.361	4.263	0.005	0.224	0.064	0.967	<0.001

**Table 4:** Subject-independent regression slopes (versus ground incline) and descriptive statistics for DMAMA by stiffness. Linear mixed-effects model to explain variations in DMAMA with the ground incline as the fixed effect and participants as the random effect. The linear fit for the effect of the ground incline on DMAMA was generated for each stiffness setting separately. The sensitivity (percent foot length per degree incline), *p*-value, and R^2^ value are shown for each linear regression.

LOW			MEDIUM			HIGH		
Sensitivity to Incline	*p*-Value	R^2^	Sensitivity to Incline	*p*-Value	R^2^	Sensitivity to Incline	*p*-Value	R^2^
0.540	0.005	0.958	0.450	0.016	0.954	0.250	0.063	0.975

**Table 5:** Correlation strength of subject-independent model to subject-dependent data. Goodness of fit when using the subject-independent sensitivity of DMAMA to the ground incline to explain individuals’ data; R^2^ values indicate how well subject-independent trends explained intra-subject variability.

		LOW	MEDIUM	HIGH
		R^2^		
Participant	1	0.343	0.153	0.026
	2	–0.006	–0.380	–0.155
	3	–0.103	–0.133	–0.078
	4	0.602	0.554	0.015

**Table 6:** Subject-dependent regression slopes (versus ground incline) and descriptive statistics for DMAMA by stiffness. Subject-dependent linear regression coefficients for the effect of the ground incline on DMAMA in each stiffness setting, using individual participant data. The linear fit for the effect of the ground incline on DMAMA is shown for each participant. The sensitivity (percent foot length per degree incline), *p*-value, and R^2^ value are shown for each linear regression.

		LOW			MEDIUM			HIGH		
		Sensitivity to Incline	*p*-Value	R^2^	Sensitivity to Incline	*p*-Value	R^2^	Sensitivity to Incline	*p*-Value	R^2^
Participant	1	0.965	<0.001	0.505	0.670	<0.001	0.328	0.535	<0.001	0.224
	2	0.322	<0.001	0.246	0.181	0.036	0.065	0.117	0.107	0.037
	3	0.214	0.035	0.066	0.187	0.029	0.068	0.169	0.023	0.071
	4	0.646	<0.001	0.630	0.699	<0.001	0.641	0.165	0.258	0.024

## Data Availability

The data presented in this study are available on request from the corresponding author.

## Abbreviations

DMAMA	Dynamic mean ankle moment arm
DOF	Degrees of freedom
DR	Down ramp
DS	Down stairs
$DMAMA_{adjusted}$	DMAMA value adjusted for random effect
$DMAMA_{average}$	Average adjusted DMAMA value
$DMAMA_{predicted}$	DMAMA value predicted by linear mixed-effects model
${\vec{F}}_{sagittal}$	Force in sagittal plane
${\bar{F}}_{sagittal}$	Magnitude of mean force in sagittal plane
${\vec{F}}_{x}$	Force in x-direction
${\vec{F}}_{y}$	Force in y-direction
${\vec{F}}_{z}$	Force in z-direction
GRF	Ground reaction force
IMU	Inertial measurement unit
I	Magnitude of ground reaction force impulse
J	Magnitude of ankle moment impulse
LC	Load cell
LDA	Linear discriminant analysis
LG	Level ground
${\vec{M}}_{ankle}$	Ankle moment
${\vec{M}}_{knee}$	Knee moment
${\vec{M}}_{sagittal}$	Moment in sagittal plane
${\bar{M}}_{sagittal}$	Mean ankle moment in sagittal plane
${\vec{M}}_{x}$	Moment in x-direction
${\vec{M}}_{y}$	Moment in y-direction
${\vec{M}}_{z}$	Moment in z-direction
${\vec{r}}_{LC}^{}$	Vector from joint center to center of load cell
${\bar{R}}_{LC}^{Shank}$	Rotation matrix from load cell coordinate system to shank coordinate system
RSS	Residual sum of squares
TSS	Total sum of squares
UR	Up ramp
US	Up stairs
VSF	Variable-stiffness foot
